# Breeding techniques to dispense higher genetic gains

**DOI:** 10.3389/fpls.2022.1076094

**Published:** 2023-01-19

**Authors:** Achala Anand, Madhumitha Subramanian, Debasish Kar

**Affiliations:** Department of Biotechnology, Ramaiah University of Applied Sciences, Bangalore, India

**Keywords:** plant breeding, CRISPR Cas-9, marker-assisted selection, genomic selection, genotypic technology

## Abstract

Plant breeding techniques encompass all the processes aimed at improving the genetic characteristics of a crop. It helps in achieving desirable characteristics like resistance to diseases and pests, tolerance to environmental stresses, higher yield and improved quality of the crop. This review article aims to describe and evaluate the current plant breeding techniques and novel methods. This qualitative review employs a comparative approach in exploring the different plant breeding techniques. Conventional plant breeding techniques were compared with modern ones to understand the advancements in plant biotechnology. Backcross breeding, mass selection, and pure-line selection were all discussed in conventional plant breeding for self-pollination and recurrent selection and hybridisation were employed for cross-pollinated crops. Modern techniques comprise of CRISPR Cas-9, high-throughput phenotyping, marker-assisted selection and genomic selection. Further, novel techniques were reviewed to gain more insight. An in-depth analysis of conventional and modern plant breeding has helped gain insight on the advantages and disadvantages of the two. Modern breeding techniques have an upper hand as they are more reliable and less time consuming. It is also more accurate as it is a genotype-based method. However, conventional breeding techniques are cost effective and require less expertise. Modern plant breeding has an upper hand as it uses genomics techniques. Techniques like QTL mapping, marker assisted breeding aid in selection of superior plants right at the seedling stage, which is impossible with conventional breeding. Unlike the conventional method, modern methods are capable of selecting recessive alleles by using different markers. Modern plant breeding is a science and therefore more reliable and accurate.

## Introduction

Plant breeding can be defined as a process wherein, specific heritable changes are induced in plants through human efforts (Orton, 2020). It is an ongoing attempt in developing plants of superior phenotypes which produce more yield, resistance to diseases and abiotic stressors, synchronous maturity etc. (Bhargava and Srivastava, 2019). The scientific and technological advancements during the 20^th^ century fastened the process of plant breeding and it was no longer just a skill, people took it up as a profession. Mendel’s laws were of utmost value during the 20^th^ century and provided a sturdy framework within which breeding was extensively practiced. Watson and Crick’s discovery of DNA as genetic material in the 1940s paved way for a novel era of biological discoveries which were appropriately incorporated by several plant breeders. During the late 20^th^ century, the Mendelian laws along with the upcoming cell and molecular biology approaches, hastened the plant breeding industry. Currently, with techniques like genome sequencing and editing, the plant breeding industry has further been improved and has seen huge profits (Orton, 2020).

Higher yield, pest resistance in crops, etc. is required to keep up with the rapid growth and demand globally for food crops. In recent times, this has been achieved using advanced plant breeding techniques. Although they are extremely reliable and consistent with their results, they are a method of artificial growth. The potential risks, drawbacks and challenges of modern breeding methods are yet to be explored.

## Conventional plant breeding

Plant breeding can be classified into two main types based on the methods and available tools, conventional and unconventional plant breeding. Conventional breeding can be defined as a process which involves the development of new varieties by using natural methods. In conventional breeding, desirable traits are put together from different gene pools but closely related by a process of cross hybridisation and therefore the product of conventional breeding is one in which pre-existing traits are mixed and matched to give rise to desirable crops (Acquaah, 2015).

Conventional plant breeding follows a particular sequence of steps. It begins with setting a clear objective, creating and assembling variation, selection, evaluation, release, multiplication and finally distribution of the new plant.

**Figure 1:**
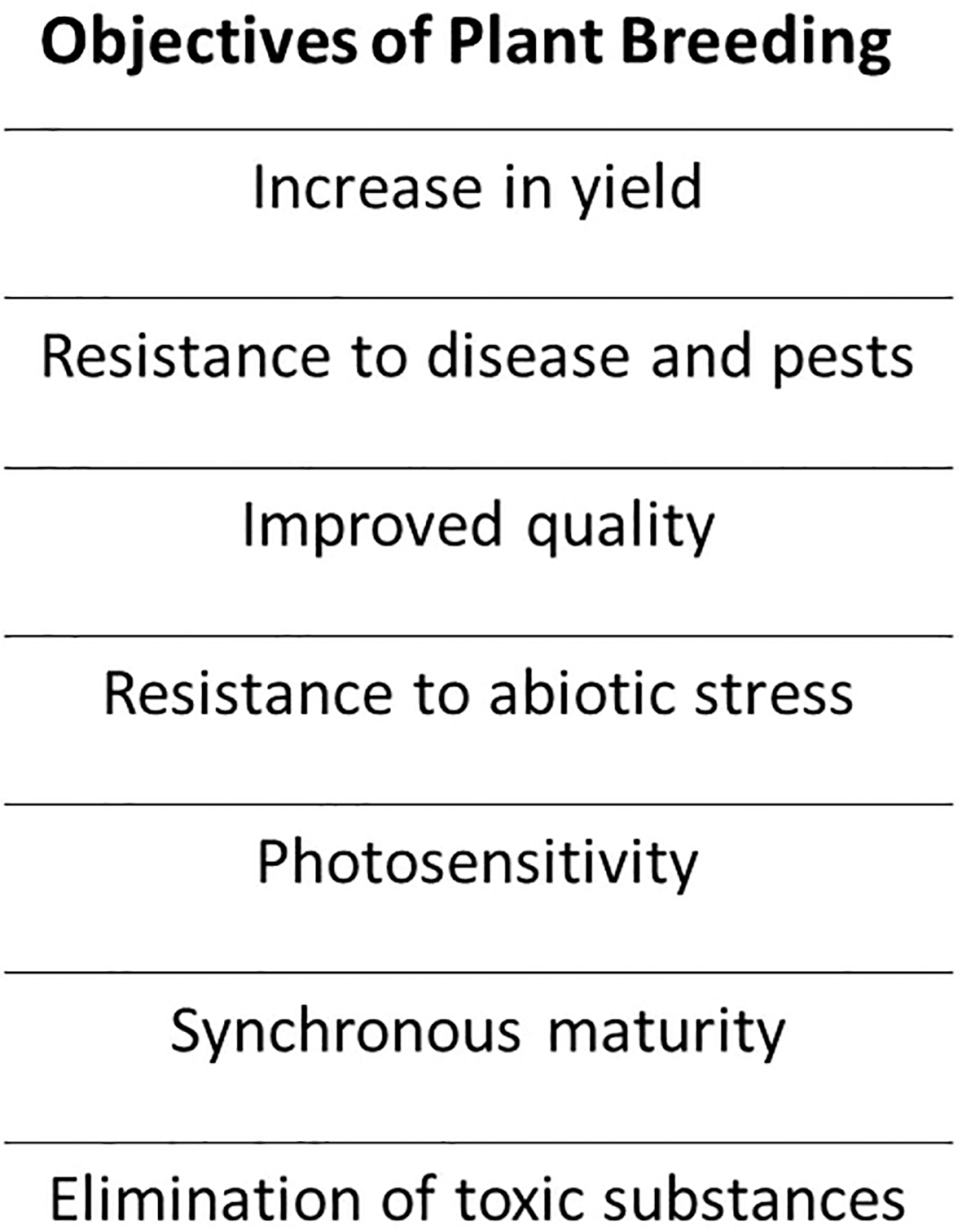
Objectives of Plant Breeding.

The species and intended application of the cultivar being created will determine the breeding goals. Prior to starting the breeding programme, the breeder must clearly identify its goals while taking the demands of end users into consideration. Thinking about it from the standpoint of cultivating the plant profitably (e.g. need for high yield, disease resistance, early maturity, lodging resistance, etc.). The processes should be taken into account when considering how effectively and affordably to use the cultivar as a source of raw materials for creating new goods (e.g. canning qualities, fibre strength, wood quality, mechanised production) (**Figure 1**).

The next stage is to gather the necessary germplasm for starting the breeding programme after deciding on the breeding objective(s). If, for instance, the goal is to breed for disease resistance, the gene causing the disease must be present when the base population is created. By artificially crossing suitable parents, the targeted gene is most frequently introduced into the base population. Breeders may try to incorporate the gene if it isn’t already present. Mutagenesis is a widely used traditional technique for generating a gene that does not exist (Acquaah, 2015).

Breeders have created standardised breeding techniques for different species based on geneti characteristics. Species can be selected or bred using techniques based on their reproductive strategies, genetics, or whether the final result should be uniform or varied. A small number of genotypes emerge from the breeding process’ final selection cycle as possible candidates for development into cultivars and release to farmers. These genotypes are put through a rigorous assessment process, which must take place in environments similar to those in which the cultivars will be produced for sale (Acquaah, 2015).

## Advances in conventional breeding technologies and techniques

Breeders engage in two fundamental tasks when developing elite cultivars: they build or assemble germplasm and then they distinguish amongst (select) variability to find and promote suitable individuals who match the breeding objectives. These two actions account for a sizable portion of a breeding program’s efficacy and efficiency. Breeders thus look for new or improved technology and procedures that support these efforts. Here are a few of the most important, each of which may also have a related approach.

### Selection

The most basic method for crop enhancement is this, and it is employed by both skilled scientists and inexperienced farmers. In essence, it is the process of selecting and advancing suitable plants by differentiating among variety.

### Artificial pollination

This kind of controlled pollination is used for a variety of purposes, including genetic research, breeding stock development, enhancing fruit set, and seed production.

### Hybridization

It entails the use of managed pollination, which may be accomplished through artificial techniques. Breeders may create hybridization blocks where controlled pollination takes place, depending on the programme.

### Wide crosses

Wide crosses for cultivated species are those that use components from outside the primary gene pool. They may entail a cross between two species or even two genera (intergeneric cross). The likelihood of genetic difficulties leading to infertility and poor success increases with the genetic distance between the parents.

### Embryo culture

Because of infertility issues, the embryos resulting from especially wide crosses do not develop normally and need to be extracted prematurely.

### Chromosome doubling

Wide interspecific crosses include parents with various chromosomal counts. Due to meiotic incompatibility, the hybrid arising from such crossings is reproductively sterile.

### Doubled haploids

When a haploid cell experiences chromosomal doubling, which can occur *in vivo* or *in vitro* and be naturally occurring or purposefully generated in plant breeding, the outcome is a doubled haploid genotype. Doubled haploid methods are currently applicable to many hundreds of plants, however the technique has benefits and drawbacks. The main drawback is that the populace cannot be forced to participate in selection. Hybrid maize breeding has successfully used doubled haploid technology.

### Bridge crossing

Bridge crossing is a method for crossing two parents with varying ploidy that is employed in broad crossings or to make a transitional or intermediate cross. The intermediate hybrid is given chromosomal doubling to make it fertile because it is reproductively sterile.

### Protoplast fusion

This method is very beneficial for wide crossing. In situations where pollination and regular fertilisation are difficult or impossible, protoplasts may be fused in a laboratory setting to produce hybridization. In order to produce potato plants that were resistant to the potato leaf roll disease, somatic fusion was employed.

## Modern plant breeding techniques

Modern plant breeding techniques came into being when molecular techniques was integrated along with conventional breeding techniques in order to achieve higher genetic gains. This was done by identifying the desired traits of the crop and their respective phenotypes and genotypes. On application of molecular techniques and genomics, the crop is enhanced.

There was a need for Modern breeding techniques due to the extensive time taken for Conventional breeding techniques. Sexual incompatibility and the sexual barriers in the form of pre and post fertilization were also major concerns (Bhargava and Srivastava, 2019). To achieve unique and more specific traits like higher absorption of nutrients, resistance to weeds, prevention of pests, decreased time of harvest, etc., conventional breeding techniques were combined with other branches of science (Lamichhane and Thapa, 2022).

In order to achieve higher genetic gains, genomic selection, enviromics and High Throughput Phenotyping (HTP) were utilised. It was also said that “Modern plant- breeding Triangle” consists of genomics, phenomics and enviromics (Crossa et al., 2017). Several staple crops like rice, wheat, sorghum and maize have successfully been bred using the above techniques (**Table 1**).

**Table 1:** Successful examples of modern plant breeding techniques.

Name of Technique	Mechanism of Technique	Example
Marker assisted selection Marker Assisted Selection (MAS)	GS works on employing DNA markers that are responsible for the expression of desired characteristics in crops	The DNA marker (9871.T7E2b) linked to the blast resistance phenotype in the presence of the Pi40 gene in a 70-kb chromosomal region was obtained from NBS-LRR disease resistance motif sequences. (Jeung et al., 2007)
Crispr Cas-9	CRISPR/Cas9 edits genes by accurately slicing DNA, which is then repaired by the body’s own mechanisms. The Cas9 enzyme and a guide RNA make up the system’s two components	CsLOB1 gene of Citrus was edited using Crispr Cas-9 to provide resistance to Citrus canker disease. (Nerkar et al., 2022)
High Throughput Phenotyping (HTP)	It is an advanced technology which generates phenotypic data of desired plant traits on utilising automated trait analysis and also involves automated sensing, data acquisition and data analysis.	SD1, Hd1, and OsGH3-2 genes of Rice (Oryza sativa L.) were modified to improve the crop yield and quality. (Xiao et al., 2022)

## Marker assisted selection

**Figure 2:**
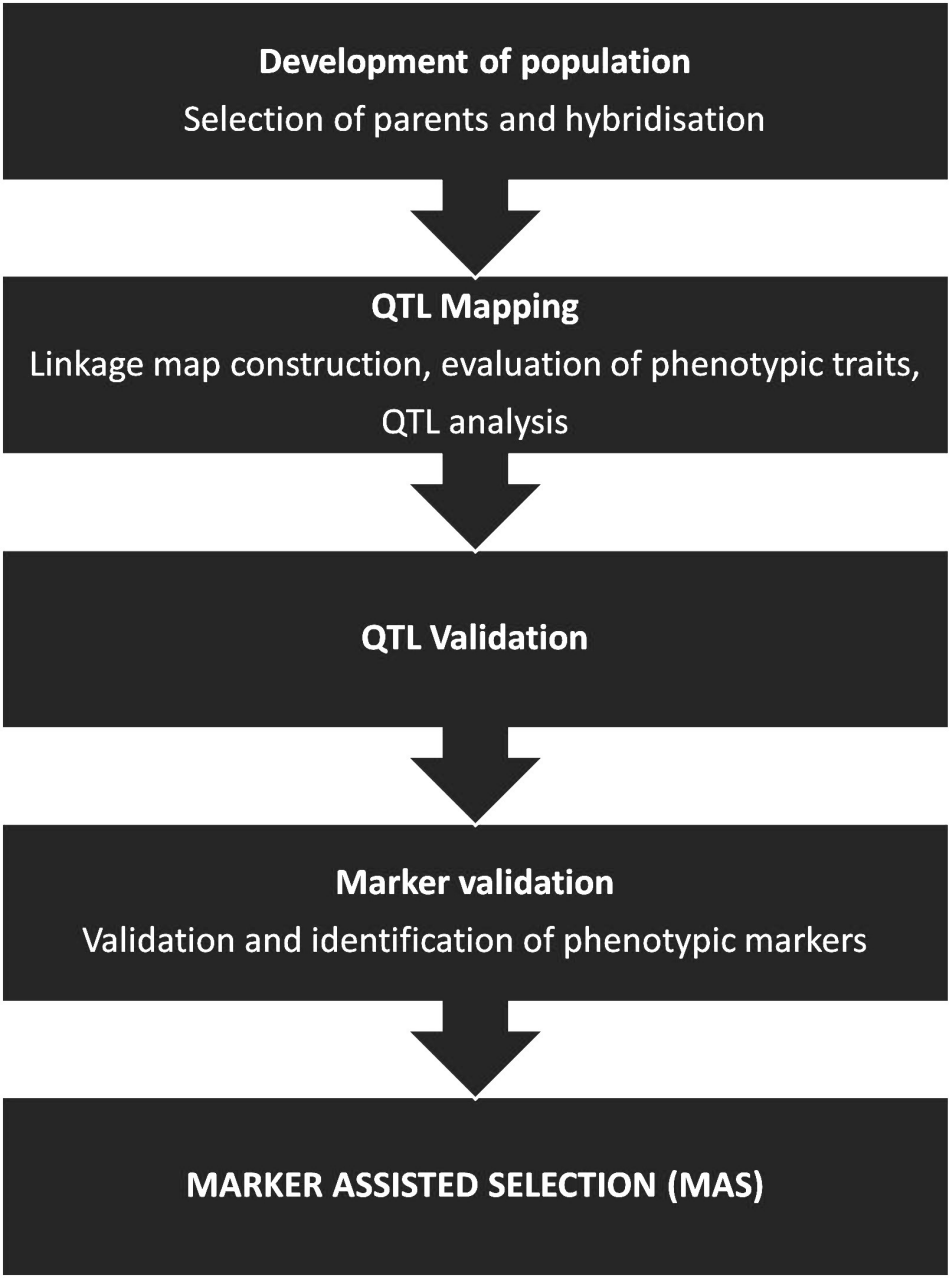
Marker development pipeline for Marker Assisted Selection (MAS).

In Marker Assisted Selection, genotypic markers are utilised in determining the phenotypic markers responsible for the desired trait with the help of bioinformatics tools. This method has proven to be much more efficient and accurate in comparison to the conventional method of direct phenotypic selection, which can be highly time consuming, more labour intensive and also less accurate. (**Figure 2**)

Markers are broadly categorised into 4 types- Morphological, Biochemical, Cytological and Molecular (DNA) based markers (Kumawat et al., 2020).

### Morphological markers

Morphological markers are phenotype- based markers. They are meant for traits that reflect the qualitative characteristics of a plant like colour of flower, shape of seed, height of plant, etc.

### Biochemical markers

Biochemical markers are commonly known as isozymes. They are multi- molecular forms of enzymes that perform the same function but are coded by non- identical genes. These markers have a property of co-dominance. They have been proved successful in several applications like detection of population structure, population subdivision, genetic diversity and gene flow, although, they don’t have a large variety and don’t identify polymorphism to a great extent.

### Cytological markers

Cytological markers are chromosome- based markers that show variations in distribution of euchromatin and heterochromatin along with position, shape, size, number and order of chromosomes. They are widely used in physical mapping.

### Molecular (DNA- based markers)

Molecular markers are most commonly used in plant breeding. These markers, not only identify the gene responsible for the desired trait, but also flag the gene such that it can be identified even in future generations. There are mainly 3 types of Molecular markers:

- Hybridization-based markers: Examples: Restriction Fragment Length Polymorphism (RFLP)
- Polymerase chain reaction (PCR)-based markers: Examples: RAPD, AFLP, SSR, chloroplast microsatellites (cpSSRs), randomly amplified microsatellite polymorphisms (RAMP), and intersimple sequence repeat (ISSR)
- Sequence-based markers: Examples: Single nucleotide polymorphism (SNP) that were developed by the introduction of the DNA sequencing technologies like next-generation sequencing (NGS) and genotyping by sequencing (GBS) resulting in high polymorphism (Bhargava and Srivastava, 2019).

## Genomic selection

In recent times, climate change has been quite drastic. Constant change in rainfall, soil acidity, increased temperature, etc. can significantly affect the growth and yield of crops.

Selection of crop variants through the method of phenotypic selection is laborious, time- consuming and does not always promise accurate results. Some crops even take a few years before they can express themselves. This method also provides room for considerable experimental error (Crossa et al., 2017).

Genomic selection uses DNA- based markers on a training population that express their genotypes as well as their phenotypes in order to predict the superior genes with desired traits.

The first a set of genotypes to be phenotyped is called as a training population, which are identified and phenotyped. A regression model is then trained to predict GEBVs for individuals which were not phenotyped. The GEBV (Genomic Estimated Breeding Values) are determined by adding the impacts of the genetic markers, or haplotypes of these markers, throughout the whole genome, that captures all quantitative trait loci (QTL) that affect any variation in trait (Ibeagha-Awemu and Khatib, 2017).

## High throughput phenotyping

The rapid expansion of the food industry has in turn brought the need for the agronomic industry to produce more crops. HTP works on the basis of phenotyping (morphological, physiological, biochemical and molecular factors) as it is the initial and more important step of plant breeding. It is an advanced technology which generates phenotypic data of desired plant traits on utilising automated trait analysis and also involves automated sensing, data acquisition and data analysis. On using this technology, there is no need to wait for a plant to mature and express itself later at its life cycle, as it can be analysed and phenotyped at its initial stage of growth (Jangra et al., 2021).

Imaging technologies are used for plant phenotyping, which is mainly carried out by electromagnetic radiation. The properties of this radiation (absorption, emission, transmission, reflection and fluorescence) reflect the health of the plant, which cannot be detected by the naked eye. The spectral information is obtained through this advanced process of imaging. The ratio of intensity of reflected light to intensity of illuminated light is measured in different wavelengths. The reflectance is high on interaction with photoactive compounds like anthocyanins and chlorophyll when compared to protein ad water- rich regions which have low reflectance. There are different types of Imaging which are widely used- Visible Light Imaging, Fluorescence Imaging, Thermal Imaging and Tomographic Imaging (Jangra et al., 2021).

## Novel plant breeding techniques

Genome editing is a technology used to manipulate genes precisely as Clustered regularly interspaced short palindromic repeats (CRISPR)/CRISPR associated protein (Cas) system. There have been some milestones in plant breeding using CRISPR Cas9 technology- i) *Solanum pimpinellifolium*, a progenitor of the tomato, may be rapidly domesticated by using CRISPR/Cas-mediated multiplex editing of “domestication genes” (e.g. loci linked to desired features). ii) Somatic homologous recombination (HR) utilising a homologous chromosome as a template can be used to repair targeted double-strand breaks (DSBs) caused by CRISPR/Cas (Rönspies et al., 2021).

## Discussions and conclusion

The benefit of traditional plant breeding is that it increases the genetic resources that may be used to enhance crops by introducing the desired features. However, certain plants run the danger of losing genetic variety and becoming vulnerable to environmental stress. Therefore, the problems with global food security cannot be solved by using traditional agricultural techniques. Several conventional and molecular methods, such as genetic selection, mutagenic breeding, somaclonal variations, whole-genome sequence-based methods, physical maps, and functional genomic tools, have been used to improve agronomic traits related to yield, quality, and resistance to biotic and abiotic stresses in crop plants. Modern plant breeding techniques has, however, been brought about by recent developments in genome editing technologies utilising programmable nucleases, clustered regularly interspaced short palindromic repeats (CRISPR), and CRISPR-associated (Cas) proteins (**Table 2**).

**Table 2:** Comparison between conventional and modern breeding techniques.

Conventional plant breeding	modern plant breeding
This breeding process places a greater emphasis on phenotype	This breeding process places a greater emphasis on genotype
It is labor- intensive and highly time consuming to develop a new hybrid	It requires lesser time, but needs more expertise to develop a new hybrid
Dominant genes are typically the only ones chosen. Recessive allele selection is a more process	Newly used technologies like Genomic selection, enviromics and High Throughput Phenotyping (HTP) Through the use of markers and the identification of particular gene locations, recessive alleles may be chosen in a very quick process
It is carried out physically by local breeders using basic tools, making it less accurate	Advanced technologies like Marker Assisted Selection (MAS), Genomic selection and High Throughput Phenotyping (HTP) are used

In the current scenario of constant development, rapid climate change, unpredictable rainfall, etc., modern breeding techniques is required to produce the crops with consistent produce. There is also a need for increased yield with the expanding food industry. Therefore, using latest and advanced technology to the power of agronomic development is more practical and sustainable.

## Author contributions

AA and MS have contributed equally to the development of this manuscript with guidance from DK. All authors contributed to the article and approved the submitted version.
